# Metal–Organic Framework (MOF)-Based Micro/Nanoscale Materials

**DOI:** 10.3390/nano15141096

**Published:** 2025-07-15

**Authors:** Jian Wang, Wenqian Chen, Pengyan Wu

**Affiliations:** 1 Jiangsu Key Laboratory of Green Synthetic Chemistry for Functional Materials, School of Chemistry and Materials Science, Jiangsu Normal University, Xuzhou 221116, China; 2 Key Laboratory of Organic Compound Pollution Control Engineering, Ministry of Education, School of Environmental and Chemical Engineering, Shanghai University, Shanghai 200444, China

This Special Issue showcases cutting-edge advances in the design, synthesis, and multifaceted applications of Metal–Organic Framework (MOF)-based micro/nanoscale materials. The research collected within highlights the remarkable versatility of these porous architectures, spanning fundamental property investigations to groundbreaking functional applications. Contributions delve into critical areas such as enhancing material stability in challenging environments (e.g., basic solutions) [1], elucidating intricate sorption mechanisms (including water-bridge-mediated pore condensation) [2,3], and leveraging computational methods like machine learning [4] and Shannon entropy [5] for property prediction and material screening, and MOF-based nanocomposites in heat exchangers [6]. Furthermore, the Issue explores the transformative potential of MOF derivatives and composites in energy storage (transition metal oxides on carbon fibers) [7], catalysis (glycerol carbonate synthesis) [8], sensitive chemical detection (acetone, VOCs, Hg^2+^) [9,10,11], electromagnetic wave dissipation [12], and innovative drug delivery systems [13], setting the stage for significant technological impacts.

We summarize the scientific contributions below.

A systematic study was conducted to assess the stability of Zr-based UiO-66 MOFs under basic conditions, addressing a critical limitation of their practical applications in catalysis and industrial processes. The authors comprehensively evaluated 11 inorganic and organic bases, correlating framework degradation with base strength (p*K*_b_) and concentration. It was revealed that strong bases (e.g., KOH, DBU) rapidly degrade UiO-66, while weaker bases like KOAc and pyridine exhibit higher tolerance. The integration of PXRD, NMR, BET, and ICP-OES data provided robust validation for structural dissolution and porosity loss. The proposed stability guidelines offer practical insights for the design of Zr-MOF applications in basic environments [1].

By studying the water absorption mechanism of isoreticular CPO-27-type Ni-MOFs, a further study effectively correlated pore size and linker hydrophobicity with hydration behavior. The authors adeptly combined water vapor sorption, FT-IR, and defect analysis via ^1^H NMR to demonstrate a transition from discrete site binding to water-bridge-mediated pore condensation. The findings elucidate how defects influence sorption in Ni_2_ (dhtp), while larger pores exhibit bulk-like water behavior. This work contributes to the rational design of MOFs for humidity-dependent applications, though hydrolytic stability remains a notable limitation. The experimental rigor and mechanistic clarity of this study are commendable [2].

A third study reported the synthesis of aluminum-based MOF-derived carbons (C-MDC and A-MDC) via the carbonization/activation of CAU-10-H and Al-fu precursors. C-MDC exhibits hydrophobic microporosity with rapid water uptake at P/P_0_~0.6, while A-MDC shows micro–mesoporous behavior. The water adsorption aligns excellently with the Do-Do model (type 5), highlighting C-MDC’s potential for humidity control. This represents a significant and innovative contribution to the design of tunable adsorbents [3].

By integrating computational screening with machine learning, a fourth study establishes a robust framework for optimizing MOFs for the purpose of methane separation. The authors effectively employed LGBM and SHAP to identify pore-limiting diameter (PLD) as the key descriptor for diffusivity and diffusion selectivity across six binary mixtures. The work stands out for its data-driven insights into separation mechanisms and practical design principles, advancing MOF optimization for clean energy applications. The approach is innovative and offers valuable guidance for future research [4].

Presenting a novel probabilistic approach, the fifth study employs Shannon entropy and Monte Carlo simulation to quantify uncertainty in key nanofluid properties (density, heat capacity, thermal conductivity, viscosity) arising from Gaussian-distributed nanoparticle volume fraction. The approach demonstrates entropy as a unified uncertainty measure, offering a significant simplification over traditional multi-moment analyses. While the methodology is robust and implemented effectively in MAPLE, the analysis is constrained to low volume fractions (≤5%) and limited input variation (CoV = 5%). This work valuably advances uncertainty quantification in nanofluidics but requires validation under broader parameter ranges for wider applicability [5].

The sixth submission, a review, rigorously examines advancements in MOF–nanocomposite applications for heat exchangers, specifically addressing thermal conductivity enhancement, fouling mitigation (through the use of hydrophobic MOFs such as ZIF-8), and facilitating scalable synthesis via microwave-assisted methodologies. The review effectively addresses critical challenges, including industrial integration costs and long-term stability under harsh conditions, while highlighting future directions like multifunctional hybrid composites. Case studies, including BASF’s energy-efficient dehumidification system, further strengthen its practical implications. Although comprehensive in scope, a deeper discussion on standardized testing protocols would enhance its contribution to the field. Overall, it offers valuable insights for advancing sustainable thermal management and merits publication [6].

The seventh submission to this Special Issue is a well-structured study on M-doped Co-MOF-derived TMO nanosheets on carbon fibers for energy storage. The authors systematically compare the effects of Zn, Mn, and Ni doping, demonstrating Ni-doped samples’ superiority in both supercapacitors (13.3 F/g at 50 mA/g) and Li-ion batteries (410.5 mAh/g at 25 mA/g). The work innovatively leverages ion exchange to enhance conductivity and capacity, offering valuable insights for multifunctional structural energy devices. The methodology is robust, and the findings advance the field effectively [7].

In the eighth contributed study, Au nanoparticles encapsulated in Ce-based MOFs provided an innovative catalytic platform for sustainable glycerol carbonate synthesis from glycerol and CO_2_. A key strengths of the study is the achievement of the highest reported TOF (78 h^−1^) under mild conditions (1.5 MPa CO_2_), enabled by synergistic Au-CeO interfaces and MgCO_3_ as an eco-friendly dehydrant. The catalysts, characterized via XRD, TEM, XPS, and physisorption, show uniform NP distribution within MOF cavities. While the yields (≤44%) require optimization, this work demonstrates significant progress in green catalysis by valorizing waste glycerol and CO_2_. Extension to Ag/Cu NPs further highlights the system’s versatility for industrial adaptation [8].

In a ninth study, breakthrough chemiresistive acetone sensing was realized via a Bi-gallate MOF/chitosan/ionic liquid composite membrane, delivering 10 ppm detection, 15 s/3 s response/recovery, high selectivity, and flexible/biocompatible properties. Its application as a non-invasive breath analyzer for diabetes diagnosis is highly relevant and the mechanistic insight it provides into the hydrogen-bonding network is valuable. While operation at 60 °C is noted, the material shows significant promise for real-time sensing applications [9].

In the tenth contribution, novel composite FGFL-B_1_—fabricated by encapsulating thioflavin T dye in MIL-125—serves as a porous fluorescence probe for VOC detection/adsorption. It exhibits exceptional selectivity: 36-fold fluorescence enhancement for THF and yellow-to-yellowish-green shift for CCl_4_. The adsorption capacities reached 655.4 mg g^−1^ (THF) and 811.2 mg g^−1^ (CCl_4_) with excellent recyclability. While surpassing conventional MOFs’ VOC response, MIL-125 dependence (vs. NH_2_-MIL-125) and untested generalizability require further study. Test strip applications demonstrated promising on-site monitoring potential [10].

In the eleventh contribution to this Special Issue, the post-synthetic modification of UiO-66-NH_2_ with 2-(Methylthio)benzaldehyde yielded sulfur-functionalized UiO-66-NSMe, enabling highly selective aqueous Hg^2+^ detection via luminescent quenching (K_sv_ = 2.5 × 10^4^ M^−1^, LOD = 20 nM). Rigorous characterization confirms 87% modification retention and S-Hg^2+^ coordination. The sensor achieved 96.1–99.5% recovery in real water and enabled rapid in situ imaging on lettuce/test paper. The conversion of Hg^2+^-loaded material into a Lewis acid catalyst enhanced resource utilization, showcasing a strategically valuable PSM approach for environmental monitoring with circular economy potential [11].

In the twelfth contribution, Co-MOF-derived nanorods were phosphate to fabricate amorphous Co@CoPx@C composites with engineered phosphorus vacancies. This design leverages synergistic dual polarization: vacancies enhance defect polarization while P-O bonds strengthen dipole polarization, coupled with interfacial polarization at Co/C/CoPx heterojunctions and conductive loss. The resultant optimal electromagnetic parameters enable exceptional performance in Co@CoPx@C-5: −55 dB reflection loss at 2.0 mm thickness and 5.5 GHz bandwidth. The vacancy-engineering strategy effectively tunes dielectric loss/impedance matching, offering novel methods for high-efficiency amorphous absorbers, though the effects of vacancy concentration on scalability require further exploration [12].

In the final submission to this Special Issue, ZIF-8 nanocarriers were used to deliver the hydrophobic telomerase inhibitor BIBR 1532, overcoming solubility and cellular delivery barriers. The synthesized BIBR 1532@ZIF-8 nanoparticles (165 nm) enhanced drug stability and enabled pH-responsive lysosomal escape, boosting nuclear accumulation. In vitro studies demonstrated significantly amplified cytotoxicity, hTERT suppression, G0/G1 arrest, and senescence versus free drug, validated through rigorous physicochemical characterization. This innovative MOF-based strategy advances oncology therapeutics, though future work requires in vivo validation and exploration of combinatorial approaches [13].

The research presented in this Special Issue vividly demonstrates the immense potential and rapidly expanding frontiers of MOF-based micro/nanoscale materials. Significant strides have been made in understanding fundamental behaviors (sorption mechanisms, stability), developing sophisticated synthesis and modification strategies (doping, post-synthetic modification, derivation to oxides/carbons), and achieving high performance in diverse applications—from advanced energy storage and efficient catalysis to ultra-sensitive chemical sensing and targeted drug delivery. Looking ahead, key challenges remain in scaling up production, further enhancing long-term stability under operational conditions, and deepening our understanding of structure–property relationships at the nanoscale.

Future directions should prioritize integrating multiple functionalities into single platforms, exploring hybrid or multi-MOF systems, advancing AI-guided design for specific applications (as exemplified in separations and EM wave dissipation), and rigorously assessing the environmental impact and lifecycle of these promising materials, particularly for large-scale implementations like heat exchangers. The journey from tailored nanoscale design to macroscopic functional integration continues to be an exciting and fruitful endeavor.
